# Lung Adenocarcinoma of Never Smokers and Smokers Harbor Differential Regions of Genetic Alteration and Exhibit Different Levels of Genomic Instability

**DOI:** 10.1371/journal.pone.0033003

**Published:** 2012-03-07

**Authors:** Kelsie L. Thu, Emily A. Vucic, Raj Chari, Wei Zhang, William W. Lockwood, John C. English, Rong Fu, Pei Wang, Ziding Feng, Calum E. MacAulay, Adi F. Gazdar, Stephen Lam, Wan L. Lam

**Affiliations:** 1 Department of Integrative Oncology, British Columbia Cancer Research Centre, Vancouver, British Columbia, Canada; 2 Department of Genetics, Harvard Medical School, Boston, Massachusetts, United States of America; 3 Hamon Center for Therapeutic Oncology Research, University of Texas Southwestern Medical Center, Dallas, Texas, United States of America; 4 Cancer Genetics Branch National Human Genome Research Institute, National Institutes of Health, Bethesda, Maryland, United States of America; 5 Department of Pathology, Vancouver General Hospital, Vancouver, British Columbia, Canada; 6 Division of Public Health Sciences, Fred Hutchinson Cancer Research Centre, Seattle, Washington, United States of America; University of Texas MD Anderson Cancer Center, United States of America

## Abstract

Recent evidence suggests that the observed clinical distinctions between lung tumors in smokers and never smokers (NS) extend beyond specific gene mutations, such as *EGFR*, *EML4-ALK*, and *KRAS*, some of which have been translated into targeted therapies. However, the molecular alterations identified thus far cannot explain all of the clinical and biological disparities observed in lung tumors of NS and smokers. To this end, we performed an unbiased genome-wide, comparative study to identify novel genomic aberrations that differ between smokers and NS.

High resolution whole genome DNA copy number profiling of 69 lung adenocarcinomas from smokers (n = 39) and NS (n = 30) revealed both global and regional disparities in the tumor genomes of these two groups. We found that NS lung tumors had a greater proportion of their genomes altered than those of smokers. Moreover, copy number gains on chromosomes 5q, 7p, and 16p occurred more frequently in NS. We validated our findings in two independently generated public datasets. Our findings provide a novel line of evidence distinguishing genetic differences between smoker and NS lung tumors, namely, that the extent of segmental genomic alterations is greater in NS tumors. Collectively, our findings provide evidence that these lung tumors are globally and genetically different, which implies they are likely driven by distinct molecular mechanisms.

## Introduction

While the majority of lung cancer cases can be attributed to tobacco smoking, up to one quarter of lung cancers arise in never smokers (NS) [1]. When considered its own disease, lung cancer in NS is the seventh leading cause of cancer death worldwide [2]. Studies have revealed that NS lung tumors are characterized by specific clinical and genetic features [2], [3], [4], [5], [6], [7], [8], [9]. NS lung cancers are more strongly associated with female gender, East Asian ethnicity and adenocarcinoma histology [2]. Molecularly, NS tumors have a significantly higher frequency of *EGFR* mutations and *EML4*-*ALK* translocations, findings that have been clinically translated for selection of targeted therapies [2], [10], [11]. Interestingly, it was recently discovered that mitochondrial DNA (mtDNA) genomes of NS harbored more alterations than those of smokers [9]. While collectively these findings provide evidence supporting the notion that NS lung tumors are driven by distinct genetic mechanisms, they cannot explain all of the clinical disparities observed in lung tumors of smokers and NS. They do however, emphasize the need to identify additional genomic aberrations that may underlie observed differences in tumor biology and provide a rationale for undertaking a global scale comparative genomic study of lung tumors arising in smokers and NS.

Previous studies have focused on locus specific genetic features or have been limited by the use of low resolution technologies lacking whole genome coverage, comparison between NS and smokers, and/or validation in external cohorts [5], [6], [7], [8], [12], [13], [14]. Therefore, to determine whether smoker and NS lung tumors are distinct on a global level, an unbiased genome-wide survey and direct comparison is warranted.

Copy number alterations (CNAs) are known contributors to tumorigenesis, and different cancer subtypes display distinct copy number profiles which are associated with distinct phenotypic and clinical features [15]. Thus, we hypothesized that lung tumor genomes of smokers and NS would exhibit disparate patterns of DNA CNAs throughout the genome that may drive the distinct clinical presentation of these tumor types. Towards this aim, we performed a global comparative analysis of copy number changes in lung cancers from smokers and NS. Since most lung cancers in NS are adenocarcinomas, we restricted our study to this type of lung cancer. We identified unique genomic features and NS-specific CNAs that were validated in two additional cohorts. These data may provide insight into the molecular basis for the differential tumor behaviors observed in the clinic.

## Methods

### Ethics statement

All tissues were collected from the Tumor Tissue Repository of the British Columbia Cancer Agency under informed, written patient consent and with approval from the University of British Columbia - BC Cancer Agency Research Ethics Board.

### Sample accrual

Both tumor and adjacent non-malignant lung tissues were accrued for 69 treatment naive lung adenocarcinoma (AC) patients undergoing surgical resection with curative intent (BCCA tumors). These included 39 smokers (current smokers (CS) at the time of diagnosis) and 30 NS (patients who had smoked fewer than 100 cigarettes in their lifetime). Tissues were fresh-frozen and underwent pathological review to confirm AC histology and absence of cancer cells in the adjacent non-malignant lung tissue. DNA was extracted using standard phenol:chloroform procedures. Each tumor was microdissected with guidance of a pathologist (JCE or AFG) to ensure >70% tumor cell content.

### *EGFR* and *KRAS* mutation screening

Genomic DNA from each tumor was sequenced to determine *KRAS* and *EGFR* mutation status by PCR amplification and product sequencing. Exons 19 and 21 and exon 2 were screened in *EGFR* and *KRAS*, respectively. PCR was performed on 50–100 ng of DNA in 25 µL reactions using the Applied Biosystems GeneAmp PCR System 9700. Applied Biosystems BigDye Terminator v3.1 cycle sequencing kit and capillary instrumentation were used to sequence PCR products. Primer sequences and PCR conditions are supplied in Table S1. A Fisher's exact test was used to assess associations of *EGFR* and *KRAS* mutation with smoking status. A p-value<0.05 was considered significant. The relationships between clinical variables (stage, gender, age, smoking) and *EGFR* and *KRAS* mutation status were assessed using Pearson correlation.

### SNP array processing

Genomic DNA from tumor and matched non-malignant lung tissues were hybridized to Affymetrix SNP 6.0 arrays according to the manufacturer's instructions. Raw CEL probe intensity files were processed using *Partek Genomics Suite Software* (Partek Incorporated, Missouri). Probe sequence, fragment length, GC content and background adjustments were applied to correct for biases in signal intensities. Copy number profiles were generated following the Copy Number, Paired Analysis Workflow such that matched non-malignant profiles were used as a copy number baseline for each respective tumor. Genomic segmentation was applied with stringent significance thresholds to identify segmental regions of CNA (gain and loss) using the following parameters: signal to noise >0.3, minimum of 50 markers per segment, p-value threshold of 10^−7^ for the statistical difference between intensities of adjacent segments, and p-value threshold of 10^−7^ for significance of deviation of intensities in tumor tissue from intensities in non-malignant lung. This stringency enabled us to confidently distinguish altered genomic segments from technical noise. Identified segments were merged using a 1 Mbp window to combine adjacent regions of copy gain or loss. All genomic mapping was based on the March 2006, hg18 genome build. The genomic coordinates for RefSeq genes (hg18) were obtained from the UCSC genome browser [16]. SNP array data for the BCCA tumors is in compliance with the MIAME guidelines and has been deposited in the Gene Expression Omnibus (GEO accession ID pending).

### Genome comparisons

For each tumor the sum of base pairs encompassed by CNAs (both gains and losses) was used to calculate the proportion of genome altered (PGA) for each tumor. Differences in PGA between tumor genomes were investigated using a two-tailed, Student's t-test assuming unequal variance. P-values<0.05 were considered significant. Tumors with PGAs in the fifth and 95^th^ percentiles were excluded to reduce the effects of outliers. A multifactor ANOVA was performed in *R* to assess the contributions of clinical (stage, gender, age, race, and smoking status) and genetic (*EGFR* and *KRAS* mutation status) variables to the observed PGA.

Segmental alterations identified in each tumor were parsed into typed copy numbers for each SNP array element, such that every array probe was scored as 1 (copy gain), 0 (copy neutral), or −1 (copy loss). Probes with similar copy number states within individual tumors were then collapsed into genomic regions across all tumor samples. The frequency of DNA gains, DNA losses, and neutral copy number were compared in smoker and NS tumor genomes using a Fisher's Exact test with a p-value<0.05 considered significant. The Fisher's Exact test was performed in *R* using a 3×2 contingency table generating a p-value for each genomic region [17]. Significant regions within 1 Mbp of each other and with the same copy number status were merged into single regions. Differentially altered regions had to have at least 15% frequency difference between smokers and NS and a frequency of at least 15% in one group if both groups showed alteration to be reported here. *ResCalc* was used to determine the functional resolution of the SNP array data given the segmentation parameters we applied (139 kbp). Thus, we only considered differentially altered regions satisfying this size threshold [18].

The *GISTIC* (Genomic Identification of Significant Targets in Cancer) algorithm was used to investigate high-level DNA alterations (defined as frequent alterations with high magnitude changes in some samples) in smokers and NS in the BCCA cohort as it had the highest resolution copy number data for mapping focal genomic events [19]. We performed *GISTIC* analysis on the segmental alteration data with the following parameters: amplification threshold = 0.848, deletion threshold = 0.737, join segment size = 50, q-value threshold of 0.05, and hg18 genome build. Regions identified in smokers and NS were then compared to determine regions of overlap and regions of difference based on the region limit boundaries defined by *GISTIC*.

### Analysis of validation datasets

Publically available microarray data with accompanying smoking status annotation was accessed to compare findings from the BCCA tumors with external cohorts. Normalized, lung adenocarcinoma Agilent 4×44 k array comparative genomic hybridization (aCGH) data generated by the Memorial Sloan Kettering Cancer Centre (MSKCC) was obtained from http://cbio.mskcc.org/Public/lung_array_data/ [20]. The MSKCC dataset was comprised of 25 current smokers and 41 NS. Copy number profiles were generated using the segmentation algorithm, *FACADE* with default parameters and a baseline distribution of 10 kbp [21]. *EGFR* and *KRAS* mutation data were also available for these tumors from the same source. Affymetrix SNP 250 K array data generated by the Database of Genotypes and Phenotypes from the Tumor Sequencing Project (TSP) were also accessed (Study Accession: Study Accession: phs000144.v1.p1). Array data for 72 current smoker and 37 NS lung adenocarcinoma and matched non-malignant tissues were processed in *Partek* with the same normalization and segmentation parameters as the BCCA tumors, except for using a 20 marker minimum for defining segments due to lower array density. PGA was calculated for each tumor in the MSKCC and TSP datasets. A Student's t-test was used to compare PGA in smoker and NS tumors with a p-value<0.05 considered significant.

Differentially altered regions in smokers and NS were identified in the MSKCC and TSP datasets applying the same strategy as that for the BCCA tumors. Minimal common regions (MCRs) of overlap for regions differentially altered in the same direction in all three datasets were mapped. Correlations between the MCRs identified were investigated in the BCCA tumors using a Pearson correlation. A multifactor ANOVA was also performed to assess relationships between each of the six MCRs discovered and the clinical and genetic variables discussed above in the BCCA tumors (stage, gender, age, smoking status, race, and *EGFR* and *KRAS* mutation status).

## Results

### Patient demographics

Lung adenocarcinoma and matched non-malignant tissue specimens were collected from 69 patients, including 39 smokers and 30 NS (Table 1). Collectively, smoker and NS patients for this comparative study were well matched for age, gender and stage of disease; however, ethnic differences existed between smokers and NS. Consistent with trends of higher incidence of lung cancer in NS among Asians compared to Caucasians, our NS cohort was significantly enriched for Asian patients (Fisher's Exact test, p = 1.3×10^−8^), while our smoker cohort was enriched for Caucasian patients. One smoker sample was from a Native American patient.

**Table 1:** Demographic data for lung adenocarcinoma tumors sampled.

Demographic		Smokers	Never Smokers
	Total	39	30
**Gender**	Male	11	7
	Female	28	23
**Age**	Range	45–78	39–86
	Average	64	70
**Ethnicity** *	Asian	2	21
	Caucasian	36	9
	First Nations	1	0
**Stage**	IA	12	8
	IB	12	9
	IIA	2	1
	IIB	7	7
	IIIA	5	4
	IIIB	0	1
	IV	1	0
**Pack Years** **	Range	2–120	0
	Average	50	0

* The proportion of individuals of Asian descent in our NS smoker cohort was significantly greater than that of smokers. However, a recent study investigating genomic differences in Western European versus East-Asian lung adenocarcinomas reported no difference in PGA [6]. Nonetheless, our validation cohorts from MSKCC and TSP were from geographically distant research centers, whose patient demographics (including race) likely differ from our own.

** One pack year is defined as smoking one package of cigarettes every day for one year.

### *EGFR* and *KRAS* mutations segregate with smoking status

**Figure 1:**
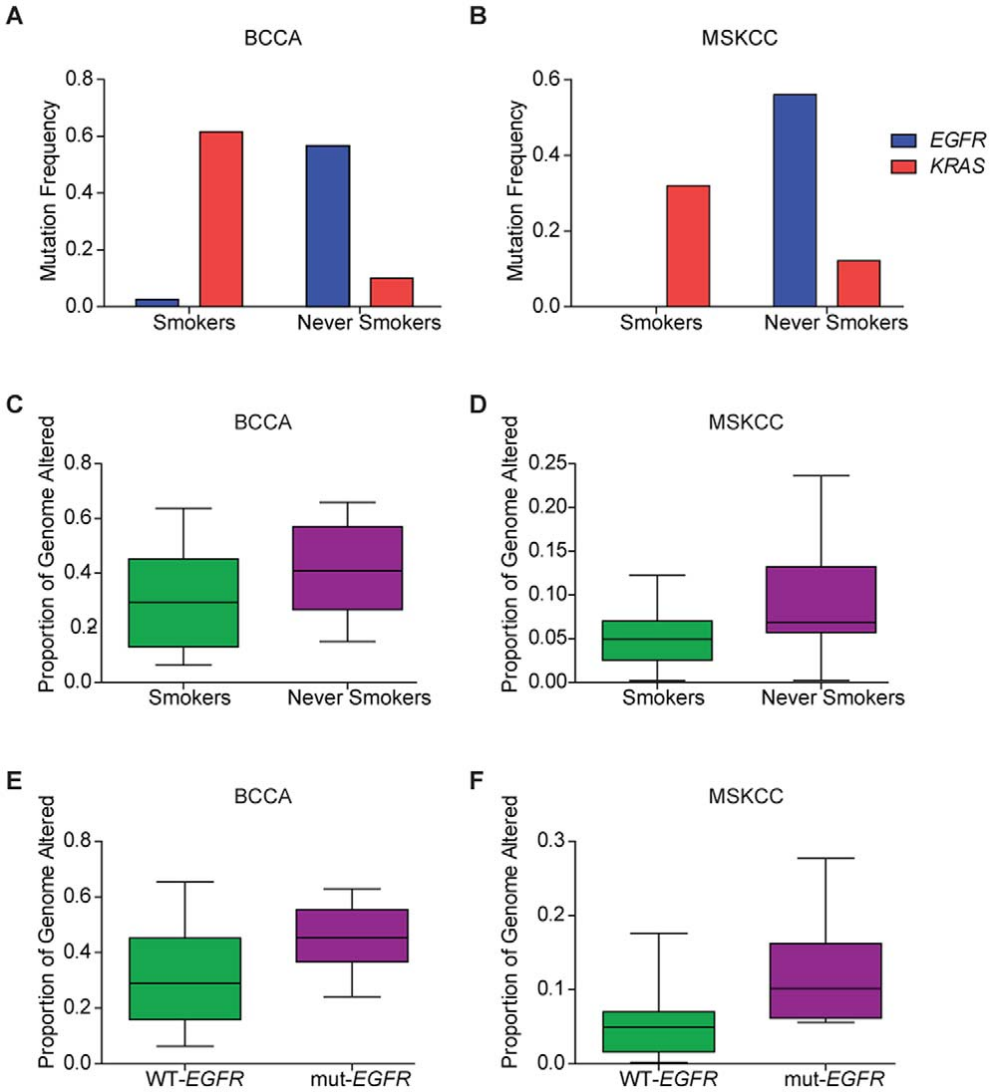
*EGFR* and *KRAS* mutation frequencies and PGA in the BCCA and MSKCC tumors. In both the BCCA (n = 69) and MSKCC (n = 66) datasets, *EGFR* and *KRAS* mutations are more prevalent in NS and smokers, respectively, consistent with the literature (A, B). The PGA in NS was greater than that of smokers in the BCCA and MSKCC tumors (C, D). PGA was also greater in *EGFR*-mutant versus *EGFR*-wild type tumors in both datasets (E,F), corroborating the difference we identified in smoker and NS tumors, as NS tumors are enriched for *EGFR* mutations.

Consistent with reported literature, mutation rates for *EGFR* and *KRAS* were associated with smoking status, confirming the accuracy of smoking history classifications. *EGFR* mutations (exons 19 and 21) were more frequent in NS (17/30 NS versus 1/39 smokers, Fisher's Exact test, p = 2.8×10^−7^) while *KRAS* mutations were more frequent in smokers (3/30 NS versus 24/39 smokers, Fisher's Exact test, p = 1.4×10^−5^) (Figure 1a). Tumors arising in Asian tumors had significantly more *EGFR* mutations than those arising in Caucasians (15/23 Asian versus 3/45 Caucasian, Fisher's Exact test, p = 5.7×10^−7^) while Caucasians had significantly more *KRAS* mutations than Asians (2/23 Asian versus 25/45 Caucasian, Fisher's Exact test, p = 1.8×10^−4^). There was no difference in mutation rates in females compared to males. A Pearson correlation analysis confirmed these associations (Table S2). *EGFR* mutations were negatively correlated with smokers (Pearson's r = −0.61) while *KRAS* mutations were positively correlated with smokers (Pearson's r = 0.52). *EGFR* mutations were also positively correlated with Asian ethnicity (Pearson's r = 0.63) and negatively correlated with *KRAS* mutations (Pearson's r = −0.48). Smoking status was also correlated with race (Pearson's r = −0.68) as our NS cohort was predominantly comprised of Asians.

*EGFR* and *KRAS* mutation data was also available for the MSKCC lung adenocarcinoma tumors; consistent with the BCCA tumors, *EGFR* mutations were more prevalent in NS (23/41 NS versus 0/25 smokers, Fisher's Exact test, p = 6.3×10^−7^) and *KRAS* mutations were more prevalent in smokers (5/41 NS versus 8/25 smokers, Fisher's Exact test, p = 0.06) (Figure 1b). There were no significant associations between *EGFR* or *KRAS* mutations and gender in the MSKCC dataset, and ethnic information was not available for analysis. In both the BCCA tumors and the MSKCC tumors, *EGFR* and *KRAS* mutations were mutually exclusive. Given the consistency of this data with the literature, we concluded that smoking histories for the BCCA and MSKCC lung tumors were accurate, ensuring we had appropriate samples to perform a smoker versus NS comparison.

### NS lung tumors have greater fractions of the genome encompassed by CNAs

**Figure 2:**
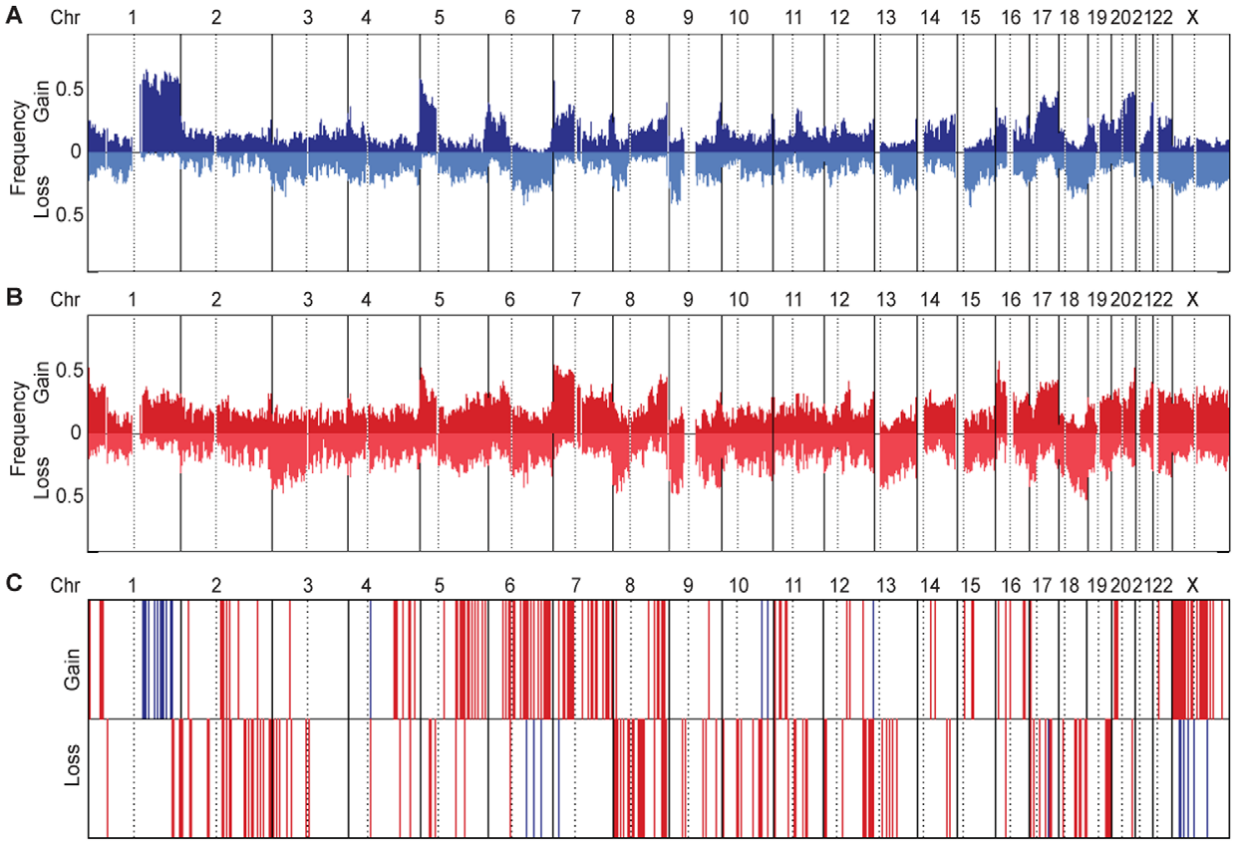
Genomic landscapes of the BCCA NS and smoker lung adenocarcinomas. The frequency of copy number alterations throughout the genome is depicted for smokers (A) and NS (B) tumors. Frequencies were calculated using a moving average window size of 500 SNP array probes. Each vertical box represents a single chromosome with the hashed line indicating the centromere. The genomic locations of the differentially altered regions (n = 313) identified in the BCCA tumors are indicated in plot C. Red bars indicate NS-specific regions and blue bars indicate smoker-specific regions.

Copy number profiles were generated for each BCCA lung tumor by performing genomic segmentation with stringent significance thresholds to ensure alterations called were non-random genetic events. All CNAs identified were somatic events as opposed to germline variants, as each profile was generated using matched non-malignant lung tissue as a reference. The frequency of CNAs throughout the genome (calculated using a moving average window of 500 SNP array probes) for the 39 smokers and 30 NS is depicted in Figure 2. Upon plotting the frequencies of alteration at each locus in the genome, we noted that the frequencies appeared to differ between smokers and NS, despite the similar distribution of CNAs throughout the genome (Table S3). To quantitatively assess this observation, we calculated the fraction of each tumor genome that was encompassed by CNAs and termed this measure Proportion of Genome Altered (PGA). Comparison of PGA between smokers and NS revealed that indeed, NS have a greater PGA than smokers (Student's t-test, p = 0.03) (Figure 1c, Table 2). Although there was no significant difference between the fraction of genomes encompassed by copy gains, NS genomes had a larger fraction affected by DNA losses than smokers (Student's t-test, p = 0.02) (Table 2). To address the possible influences of the mutation and ethnic imbalances in our cohort, PGA in *EGFR* mutants (n = 17) versus *EGFR* wild-types (n = 13) and Asians (n = 21) versus non-Asians (n = 9) in NS tumors only were compared. There was no significant difference in PGA between mutant and wild-type *EGFR* NS tumors (Student's t-test, p = 0.21). There was also no significant difference in PGA in Asian versus non-Asian NS tumors (Student's t-test, p = 0.06).

**Table 2:** Proportion of genome altered in smokers and never smokers.

PGA Type	Measure	Combined	Smokers	Never Smokers
**Copy Gains**	Average	0.19	0.17	0.21
	Median	0.19	0.17	0.22
	Range	0.02–0.36	0.03–0.35	0.02–0.36
**Copy Losses**	Average	0.17	0.15	0.20
	Median	0.16	0.14	0.20
	Range	0–0.43	0–0.32	0–0.43
**All Copy Number Alterations**	Average	0.36	0.32	0.41
	Median	0.37	0.29	0.41
	Range	0.02–0.72	0.03–0.66	0.02–0.72

### Smoking status is the most strongly associated clinical variable with observed PGA

To further determine whether the observed genome differences were associated with clinical parameters other than smoking status and to account for the ethnic bias in our cohort, we investigated the PGA in tumors as a function of all clinical and genetic variables available for our BCCA tumors (stage, gender, age, smoking history, race, and *EGFR* and *KRAS* mutation status). A multivariate analysis revealed that smoking status explained the greatest amount of variance observed in PGA (multifactor ANOVA, F = 3.64, p = 0.06) compared to all other factors, followed by *EGFR* mutation (F = 2.49, p = 0.12) (Table S4). Since NS tumors often harbor *EGFR* mutations this finding was not surprising. We also performed a multivariate analysis in NS tumors only, and found that *EGFR* mutations explained the greatest amount of variance in the NS tumors alone (multifactor ANOVA, F = 3.22, p = 0.09) (Table S5), although it was less significant than the association of smoking status and PGA in the entire tumor dataset.

### PGA is greater in NS than smokers in additional cohorts of lung adenocarcinoma

To verify our observations were not limited to the BCCA tumors alone, we investigated publically available data for smoker and NS lung adenocarcinomas from the MSKCC and lung TSP, respectively [20], [22]. Genomic profiling in these studies was performed using aCGH (MSKCC) and SNP arrays (TSP). Thus, we employed platform-appropriate methodologies to generate copy number profiles for each tumor. In the MSKCC dataset, for which *EGFR* and *KRAS* mutation frequencies were consistent with smoking status classifications, we observed the same global pattern of CNAs; NS had greater PGA than smokers (Student's t-test, p = 4×10^−3^), validating our findings (Figure 1d). NS tumor genomes had a larger fraction affected by DNA gains (Student's t-test, p = 0.01) and DNA losses (Student's t-test, p = 0.01) than smokers. *EGFR* mutant tumors also exhibited greater PGA than *EGFR* wild-type tumors (Student's t-test, p = 3×10^−4^), consistent with our observations in the BCCA tumors.

We also interrogated the TSP dataset as an additional validation cohort of lung adenocarcinomas from smokers and NS, albeit acknowledging we were unable to confirm smoking histories with mutation information. Although it did not meet statistical significance, PGA in the TSP tumors was higher in NS than in smokers (Student's t-test, p = 0.13) (Figure S1). The reproducibility of our results in two additional datasets that were derived using different genomic profiling platforms, from independent research centers is evidence for our finding that large scale differences in genomic landscapes exist between smoker and NS tumors. Moreover, consistent with our results, *EGFR* mutations were also associated with higher PGA (Figures 1e–f), which was expected given that NS lung tumors and *EGFR* mutations are highly correlated.

### Genomic alterations common to smokers and NS

**Figure 3:**
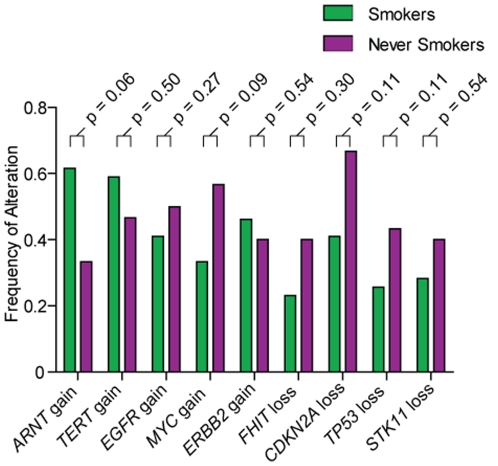
Genes commonly disrupted in smokers and NS. Frequencies of copy number alteration for 9 genes commonly reported as disrupted in lung adenocarcinoma are shown. The difference in frequencies of alteration in smokers and NS was not statistically different for any of these genes, suggesting they are equally important in lung adenocarcinoma tumorigenesis in both smokers and NS. Fisher's exact test p-values for the comparison of alteration frequencies in NS and smokers are indicated for each gene.

We observed that overall, the global distribution of copy number events identified in smokers and NS in our cohort were similar and consistent with those previously described in the literature [6], [7], [8], [20], [22], [23], [24]. For example, recurrent copy number gains on chromosomes 1q, 5p, 7p, 8q and 17q were prominent in both groups (frequency >30% in smokers and NS) and included known oncogenes such as *ARNT*, *TERT*, *EGFR*, *MYC*, and *ERBB2* (Figure 3). We observed concurrent mutation and copy number gains of *EGFR* in 10 of 17 NS with *EGFR* mutation and in the one smoker with an *EGFR* mutation, consistent with previous reports of mutation accompanied by DNA amplification [25]. However, there was no significant difference in the occurrence of *EGFR* copy number gains in *EGFR* mutant versus wild-type tumors (Fisher's Exact test, p = 0.17). Common regions of copy number loss (frequency >20% in both groups) included chromosomes 3p, 6q, 8p, 9p, 17p and 19p, and encompassed known tumor suppressors including *FHIT*, *CDKN2A*, *TP53*, and *LKB1* (Figure 3). A Fisher's Exact test to compare alteration frequencies of these known genes in smokers and NS revealed that none were significantly different between the two groups. The similarity in disruption to common lung adenocarcinoma genes in smokers and NS highlights the need to identify novel genomic aberrations that underlie the distinct clinical phenotypes exhibited by smokers and NS.

### High-level DNA alteration patterns in smokers and NS

We next sought to determine whether the high-level DNA alteration profiles of smokers and NS differed. We used the *GISTIC* algorithm to identify significant regions of focal DNA amplification and deletion [19]. Using this approach we identified 107 events in smokers and 50 events in NS (Tables S6 and S7, respectively). These findings suggest that although NS have a greater PGA, the lung tumor genomes of smokers harbor more high-level DNA alterations than NS. A total of 27 regions overlapped in smokers and NS. Of these, 13 were altered in the same direction (amplifications at 5p15.32, 7q11.21, 8p11.1, and 19q12; and deletions at 3p12.3, 5q31.3, 6q12, 6q16.1, 9p21.3, 10q21.1, 11q14.3, 19q12, and 22q13.31) while 14 were altered in opposite directions in smokers and NS (7q11.21, 8p11.1, 19q12, 2q33.3, 5q31.3, 7p15.2, 8p23.3, 8q24.3, 9p21.3, 9q33.3, 10q21.1, 16q24.1, 18q23,19q12). Some regions were large enough that two individual regions in tumors with the opposite smoking status mapped within them, producing both same and opposite direction alterations for some cytobands (7q11.21, 8p11.1, 19q12, 5q31.3, 9p21.3, 10q21.1 and 19q12). High-level DNA amplifications at 5p15.32 and 19q12 and deletion at 9p21.3 have been reported in lung adenocarcinoma previously [22]. The remaining alterations identified were specific to smokers or NS (Tables S6 and S7).

### Differentially altered regions between NS and smoker lung tumor genomes

In addition to observing a global difference in PGA between NS and smokers and differences in high-level alteration profiles, several genomic regions were found to have different alteration frequencies between the groups. For example, copy number gains of chromosome 1q were more frequent in smokers, while gains on 5q, 7p, 16p and chromosome X were more frequent in NS (Figure 2). Additionally, chromosome 3p, 8p, 13q, 17p and 19q losses were more prevalent in NS. To investigate these regions using a statistical approach, we collapsed the smoker and NS genomes into discrete regions and compared the frequencies of alteration. We identified 313 genomic regions spanning chromosomes 1–22 and chromosome X that met our criteria for differential alteration status in the BCCA tumors. These regions included both gains and losses specific to NS and smokers.

### Concordance of differentially altered regions across multiple cohorts

To identify regions of concordance across multiple datasets, we looked for differentially altered regions in the MSKCC tumors that overlapped with those identified in our BCCA dataset. Of the 68 regions that were differentially altered in the MSKCC data set between smokers and NS, our analysis revealed 21 distinct regions, spanning 9 different chromosomes that overlapped with those identified in our BCCA tumors. Three regions, all copy number gains on chromosome 1q, were specific to smokers while the remaining 18 regions included 9 copy number gains and 9 copy number losses, were specific to NS. The discrepancy in number of regions identified within each dataset is likely due to the higher resolution and better detection ability of the SNP array platform used for the BCCA analysis. Nevertheless, validation of the BCCA regions in an external dataset with well documented smoking histories demonstrates that in addition to exhibiting global differences in copy number patterns, smokers and NS exhibit regional genomic differences as well. A list of the minimal common regions (MCRs) of differential alteration shared by the BCCA and MSKCC lung tumors is provided in Table 3.

**Table 3:** Minimal common regions of differential alteration between smokers and never smokers in BCCA and MSKCC datasets.

Region	Cytoband	Chr	Start	End	Size	Status
R1	1q21.1	1	143628352	143921538	293186	Gain in Smokers
R2	1q21.1	1	146866582	146938873	72291	Gain in Smokers
R3	1q21.3	1	149678259	151582433	1904174	Gain in Smokers
R4	5q33.3	5	158495214	158674554	179340	Gain in NS
R5	5q34	5	162876149	165700950	2824801	Gain in NS
R6	7p21.2	7	13416032	13719486	303454	Gain in NS
R7	7p15.3-p15.2	7	24876559	25666836	790277	Gain in NS
R8	7p14.1	7	40005232	41936324	1931092	Gain in NS
R9	7p12.3	7	46603499	46892619	289120	Gain in NS
R10	7p12.3	7	48447956	49667305	1219349	Gain in NS
R11	8p23.1	8	9933149	10550456	617307	Loss in NS
R12	8p21.2	8	25009640	25349074	339434	Loss in NS
R13	8p21.1	8	28103525	29457984	1354459	Loss in NS
R14	8p12	8	38180851	38440527	259676	Loss in NS
R15	9q33.2	9	122816481	122966826	150345	Loss in NS
R16	10q11.21	10	42955951	43010057	54106	Loss in NS
R17	13q12.11	13	18601703	18653178	51475	Loss in NS
R18	16p13.3-p13.2	16	5278276	8866443	3588167	Gain in NS
R19	16p12.1-p11.2	16	27313417	27917742	604325	Gain in NS
R20	18q21.2	18	46480704	46480763	59	Loss in NS
R21	18q22.3	18	69959421	69976724	17303	Loss in NS

We also applied this comparison to an additional cohort (TSP) to identify the most prominent differentially altered regions between NS and smokers in the combined datasets acknowledging that this stringent criteria may increase the number of false-negative regions. This analysis revealed six MCRs concordant in all three independent datasets. All of these regions were copy number gains specific to NS, and included two regions on chromosome 5q, three regions on chromosome 7p, and one region on chromosome 16p, which encompassed a total of 13 genes (Table 4, Figure S2). The smallest MCR defined was the region at 5q33.3 which was 179 kbp in size, while the largest MCR defined was 16p13.3-13.2 which was 3.6 Mbp. Interestingly, the frequency of copy number gains at 5q33.3 and 5q34 in smokers was zero in every dataset. Gains on chromosome 7p (7p14.1 and 7p12.3) were on average 20% greater in NS than in smokers across the three datasets, as were gains at 16p13.3-13.2. The NS-associated lung cancer oncogene *EGFR* did not map to the MCRs on chromosome 7p; however, we observed a significant association between both 7p12.3 DNA gains and *EGFR* gains and mutations (Fisher's Exact test, p = 0.002 and p = 0.010). This finding is consistent with the fact that EGFR function can be influenced by multiple genetic mechanisms.

**Table 4:** Minimal common regions of differential alteration between smokers and never smokers in three independent datasets.

Region	Cytoband	Chr	Start	End	Size	Status	Genes in MCR
Region 1	5q33.3	5	158495214	158674554	179340	Gain NS	*IL12B, RNF145, UBLCP1*
Region 2	5q34	5	162876149	165700950	2824801	Gain NS	*MAT2B*
Region 3	7p14.1	7	40455608	41936324	1480716	Gain NS	*C7ORF10, INHBA*
Region 4	7p12.3	7	46603499	46892619	289120	Gain NS	*-*
Region 5	7p12.3	7	48447956	49667305	1219349	Gain NS	*ABCA13*
Region 6	16p13.3-13.2	16	5278276	8866443	3588167	Gain NS	*A2BP1, ABAT, C16ORF68, CARHSP1, PMM2, TMEM186*

### Assessing co-occurrence of identified MCRs

To assess whether the MCRs identified were independent events within our tumors, we sought to investigate whether any of the MCRs we identified were correlated with one another in the BCCA tumors (Table S8). Employing a Pearson's correlation analysis, we discovered that the three regions located on chromosome 7p were highly correlated (Pearson's r >0.78). The region on 7p14.1 was 4.7 Mbp telomeric of the first 7p12.3 region which itself was 1.6 Mbp telomeric of the second 7p12.3 region. We also observed a positive correlation between the 5q33.3 and 5q34 regions, which were located 4.2 Mbp from each other (Pearson's r = 0.64). The proximity of these intrachromosomal alterations likely contributes to the correlations observed, and these correlations indicate that the alterations on 7p may not be independent DNA gain events and likewise for the alterations on 5q; the alterations detected may actually represent one copy number gain event spanning a contiguous region involving each of the individual alterations. In addition to these intra-chromosomal associations, we discovered a positive inter-chromosomal correlation between the 5q33.3 and 16p13.3-13.2 regions (Pearson's r = 0.52), suggesting these regions are gained concurrently in some tumors.

### Multivariate analysis assessing associations of clinical features with MCRs

We next asked whether any of the MCRs we identified could explain the variance in PGA that we observed. None of the six MCRs contributed significantly to the observed PGA across the BCCA tumor dataset. Lastly, although we identified these regions by comparing smokers and NS, we performed a multivariate analysis to confirm that smoking status was the factor most strongly linked with each of the MCRs we identified. As discussed, given the highly pronounced association between *EGFR* mutations and smoking status, we expected to see that smoking history and *EGFR* mutation would account for the greatest amount of variance observed for each region. Indeed, smoking status and *EGFR* were most strongly associated with all of the regions we discovered, except for 7p14.1, for which the factor explaining the greatest amount of variance was age (followed closely by *EGFR* mutation).

## Discussion

Given the enormous efforts put forth to promote smoking cessation and prevention initiatives, in the next few decades NS (and former smokers) will constitute a larger proportion of the lung cancer population [2]. It is a well established concept that lung tumors in smokers and NS are distinct disease entities [2], [3], [26], [27]. At the DNA level, molecular differences discovered to date are gene-specific and cannot account for all of the clinical differences exhibited by smokers and NS [2], [3], [26]. In this study we sought to elucidate global genomic differences in lung adenocarcinomas from NS and smokers. Using a genome-wide comparison approach, we discovered that NS lung tumors have a greater proportion of their genomes altered than those of smokers, we identified regional genomic disparities in the tumor genomes of these two groups, and we validated our findings in two independent external cohorts from the Memorial Sloan Kettering Cancer Centre (MSKCC) and Tumor Sequencing Project (TSP).

*EGFR* and *KRAS* mutations in the BCCA and MSKCC tumors segregated with NS and smokers, respectively, consistent with the reported literature. This confirmed the accuracy of our smoking status classifications and validated that these tumors were appropriate to perform a smoker versus NS genome comparison. Genes and regions known to be frequently disrupted in lung adenocarcinoma were not preferentially disrupted in smokers or NS, and the recurrent alterations we observed in both groups were highly concordant with recent reports [6], [7], [8], [20], [22], [23], [24]. For example, the most frequently altered region detected in our 69 tumors was gain of 5p15.32-15.33 (51% of tumors), which harbors the hallmark cancer gene *TERT*. Gain of 5p was also the most common genomic alteration observed by Weir *et al.* in a collection of over 350 lung adenocarcinoma tumors [22]. Having established that regions commonly altered in lung adenocarcinoma were not associated with smoking status, we proceeded to determine whether distinct genomic features exist that may underlie the disparate clinical phenotypes observed in smoker and NS lung cancer patients.

Intriguingly, our comparative study revealed that NS lung tumors have a greater fraction of the genome encompassed by genomic alterations. Despite the caveat that our smoker and NS groups were not balanced for ethnicity or mutation (which is not surprising given the known clinical and molecular features associated with smoking status) our multivariate analysis suggested smoking history was the clinical variable most strongly associated with this observed difference. However, since an earlier study that sampled a small fraction of the genome had suggested a greater degree of alterations in smoker tumors [8], we assessed the repeatability of our results in two additional independent cohorts. Specifically we investigated whether the observed global genomic distinction in NS tumors was also evident in these two independent cohorts (MSKCC and TSP, from distant geographical sites, with likely different demographics). Across these three independently performed genomic analyses, we found corroborating results. Even though our observation held true in independent datasets, we are mindful of the fact that the contributions of mutational and smoking status cannot be distinguished in our study. It remains a possibility that PGA is associated with mutation, as PGA between NS and smokers with no mutations was not significantly different. Our study is not powered to test adjusted effects of smoking or mutation type on PGA adjusting for all other confounding factors. This is because smoking and race are correlated with *EGFR* or *KRAS* mutation. A clean comparison would require large numbers of patients in each smoking/mutation/race combination, at least 300 subjects for each, in order to achieve 80% power to detect a 10% difference in PGA at a significance level of 0.05.

Amidst the genomic instability observed in the lung adenocarcinoma tumors, we identified frequent genomic alterations whose recurrent nature signifies their selection in tumor genomes. After cataloguing numerous differentially altered regions in our dataset, we interrogated two independent cohorts to validate our findings and to reveal the most robust and pronounced regional differences in smokers and NS. We identified six MCRs of copy number gain on chromosomes 5q, 7p and 16p. It is possible that additional, less prominent MCRs, may have been identified had we used less stringent concordance criteria between the three datasets. The regions we have reported are the most robust as they are present in multiple independent cohorts. Again, we performed a multivariate analysis to confirm that smoking status (and subsequently *EGFR* mutation) was the strongest factor associated with genomic regions of difference identified. Broet *et al.* recently reported regions differentially altered in East-Asian and Western European lung adenocarcinoma tumors, none of which overlapped with the smoking-related regions we identified, indicating that our regions are not ethnic specific [6]. The two MCRs we identified on 5q were strongly correlated with one another, as were the three MCRs on 7p, suggesting they may actually be the result of single copy number events. The positive correlation we observed between 5q and 16p could signify that concurrent gains of these regions is non-random and may be biologically relevant.

A recent study profiled 60 NS lung adenocarcinomas using array comparative genomic hybridization (aCGH), albeit without a comparison against tumors from smokers, reporting several MCRs of copy number gain and loss [7]. We cross referenced our differentially altered regions to the regions identified by Job *et al.* to determine whether any of their regions might be NS-specific. Most of the regions reported by Job *et al.* were commonly disrupted in NS and smokers in our BCCA tumors; however, Job *et al.* also reported regions of copy number gain on chromosomes 5q, 7p and 16p which we identified as NS-specific. Both of our groups observed gains of chromosome 1q21 in NS, however in our cohort, 1q21.1 gain was up to 30% more frequent in smokers than NS, suggesting it may be a smoker-specific alteration.

Early profiling studies led to the discovery that gains of chromosome 16p are more common in NS than smokers, and this remains one of the few consistently replicated NS-specific genetic alterations discovered to date, which implicates the importance of this region in NS tumor biology [2], [3], [6], [7], [26]. We and others also observed an association between gain of 16p13.3-13.2 and Asian ethnicity, however, this could reflect the fact that a large fraction of NS lung cancer patients are of Asian descent [2], [6]. The earliest NS lung tumor profiling studies did not identify frequent gains of 5q in NS; however, we found two robust regions of gain at 5q33.3 and 5q34. Other recent studies have also identified frequent gains on 5q in NS lung adenocarcinomas, corroborating our results [6], [7], [8].

Our analysis revealed three distinct MCRs of gain on 7p, however none encompassed the lung cancer oncogene, *EGFR*, located on chromosome 7p11.2. The closest region (7p12.3) was situated 5.4 Mbp telomeric of the *EGFR* locus. The presence of these MCRs could imply that additional oncogenes are responsible for 7p gains, as previously suggested [28]. Investigators from the MSKCC discovered that *DUSP4*, a gene located on chromosome 8p12, was down regulated and associated with *EGFR* mutation [20]. We analyzed *DUSP4* in the BCCA dataset and confirmed this association (Fisher's Exact test, p = 0.05). Interestingly, we also mapped an MCR of loss on 8p in the BCCA and MSKCC datasets which encompassed *DUSP4* and found that this region was more frequently lost in NS tumors. Although 8p was not one of our most robust differentially altered regions, it appears genomic loss of *DUSP4* is associated with *EGFR* mutation and NS.

While many known regions of copy number alteration in lung adenocarcinoma were present in both our smoker and NS cohorts, our results, along with the well established differences in mutational profiles and clinical features, suggest lung tumors of smokers and NS develop through different molecular mechanisms. This may be similar to what has been observed in ovarian cancer, where Type I serous ovarian cancers are typically chromosomally stable and harbor mutations in the Ras signaling pathway, while high-grade serous ovarian cancers (Type II) are *RAS* wild-type and exhibit widespread copy number aberrations [29]. Intriguingly, Sidransky and co-workers recently discovered that NS lung adenocarcinoma genomes have a greater number of mitochondrial DNA alterations than smokers [9]. This finding is consistent with our discovery, providing additional evidence to support the concept that lung cancers in smokers and NS are driven by different molecular alterations. We postulate that NS lung tumors acquire specific genetic alterations early in tumorigenesis that compromise genome integrity. For example, we hypothesize that NS could be inherently predisposed to genomic instability, or they could be exposed to non-tobacco related carcinogens that drive genomic instability. Elucidation of the precise mechanism driving this instability phenotype could potentially lead to targeted therapy for NS patients, or to identify NS at risk of lung cancer development.

It is well known that the mutation profile of NS lung adenocarcinoma is distinct from that of smokers [2]. The recent discovery of increased mtDNA mutations and mtDNA content in NS relative to smokers further supports the concept that the distinction between smoker and NS tumors extends beyond *EGFR* and *KRAS* mutations [9]. Our findings provide a third and novel line of evidence towards genetic differences between smoker and NS lung tumors, namely, that the extent of segmental genomic alterations is greater in NS tumors. Collectively, our findings provide evidence that these lung tumors are globally and genetically different, which implies they are likely driven by distinct molecular mechanisms. Although the biological mechanism underlying our observations in NS remains unknown, elucidation of this mechanism is crucial to the early detection and possibly treatment of these patients, as no known risk factors or molecular features exist to assess lung cancer risk in NS besides family history. Our work provides a rationale for the stratification of patients based on smoking status in future studies, which will in turn facilitate discoveries of the nature of lung cancer in both smokers and NS. Prospective findings will have significant implications and may lead to the development of clinical tools that could be utilized to improve the prognosis of both smoker and NS patients.

## Supporting Information

Figure S1 **PGA in smokers and NS in the TSP dataset.** Although it did not meet statistical significance, NS lung tumors have greater PGA than smoker lung tumors on average.
(TIF)

Figure S2 **Six minimal common regions (MCRs) of difference between smokers and NS.** The six regions described in Table 4 are illustrated here. The region from each dataset involved in the MCR is shown with the genomic coordinates flanking each region (A). Hashed lines indicate the MCR region boundaries. Regions are not drawn to scale. The frequencies of DNA copy number gains in smoker and NS tumors for each dataset is indicated (B). Since the differentially altered regions in each dataset were defined by merging adjacent significant regions into one (as described in the Methods), the frequencies illustrated are the minimum frequencies observed for regions contributing to the merged region. Fisher's exact test p-values for the comparison of alteration frequencies in NS and smokers are indicated for each region in each dataset.
(TIF)

Table S1 **Genomic DNA PCR primers.** PCR on genomic DNA was performed to determine *EGFR* and *KRAS* mutation status in the BCCA lung tumor cohort. Mutations and primer sequences are shown.
(DOC)

Table S2 **Matrix summarizing correlations between clinical and genetic variables in the 69 BCCA tumors.** A correlation analysis was performed to determine the associations between different clinical and molecular factors in the BCCA lung tumor cohort. Correlation coefficients for each pair of variables are shown.
(DOC)

Table S3 **Summary of alteration frequencies in BCCA smoker (n = 39) and NS (n = 30) lung tumors.** The frequency of copy number alterations throughout the genome (calculated using a moving average window of 500 SNP array probes) was determined and summarized for each group (all 69 tumors, 39 smoker tumors and 30 never smoker tumors). The minimum (Min), maximum (Max), median, and average frequencies for each group are indicated. Total frequency (Gain & Loss), frequency of gain, and frequency of loss are reported.
(DOC)

Table S4 **Multifactor ANOVA test results for assessing the effects of clinical and genetic factors on observed PGA in 69 BCCA tumors.** A multifactor ANOVA was performed to investigate the effects of multiple factors on PGA in the BCCA lung tumor cohort (n = 69). The ANOVA test statistics are shown.
(DOC)

Table S5 **Multifactor ANOVA test results for assessing the effects of clinical and genetic factors on observed PGA in NS tumors only.** A multifactor ANOVA was performed to investigate the effects of multiple factors on PGA in never smokers only (n = 30). The ANOVA test statistics are shown.
(DOC)

Table S6 **High-level DNA changes in smoker lung tumors.** *GISTIC* was used to reveal high level DNA alterations in 39 smoker lung tumors from the BCCA. Copy number status, genomic location, and frequency of alteration are indicated.
(DOC)

Table S7 **High-level DNA changes in NS lung tumors.** *GISTIC* was used to reveal high level DNA alterations in 30 never smoker lung tumors from the BCCA. Copy number status, genomic location, and frequency of alteration are indicated.
(DOC)

Table S8 **Matrix summarizing correlations between each minimal common region identified in the 69 BCCA tumors.** The six minimal common regions identified in the BCCA tumor cohort were assessed for their correlations with one another. The correlation coefficients for each pair of regions are shown.
(DOC)

## References

[1] Parkin DM, Bray F, Ferlay J, Pisani P (2005). Global cancer statistics, 2002.. CA Cancer J Clin.

[2] Sun S, Schiller JH, Gazdar AF (2007). Lung cancer in never smokers–a different disease.. Nat Rev Cancer.

[3] Subramanian J, Govindan R (2007). Lung cancer in never smokers: a review.. J Clin Oncol.

[4] Pleasance ED, Stephens PJ, O'Meara S, McBride DJ, Meynert A (2010). A small-cell lung cancer genome with complex signatures of tobacco exposure.. Nature.

[5] Sanchez-Cespedes M, Ahrendt SA, Piantadosi S, Rosell R, Monzo M (2001). Chromosomal alterations in lung adenocarcinoma from smokers and nonsmokers.. Cancer Res.

[6] Broet P, Dalmasso C, Tan EH, Alifano M, Zhang S (2011). Genomic profiles specific to patient ethnicity in lung adenocarcinoma.. Clin Cancer Res.

[7] Job B, Bernheim A, Beau-Faller M, Camilleri-Broet S, Girard P (2010). Genomic aberrations in lung adenocarcinoma in never smokers.. PLoS One.

[8] Massion PP, Zou Y, Chen H, Jiang A, Coulson P (2008). Smoking-related genomic signatures in non-small cell lung cancer.. Am J Respir Crit Care Med.

[9] Dasgupta S, Soudry E, Mukhopadhyay N, Shao C, Yee J (2011). Mitochondrial DNA mutations in respiratory complex-I in never-smoker lung cancer patients contribute to lung cancer progression and associated with EGFR gene mutation.. J Cell Physiol.

[10] Pao W, Girard N (2011). New driver mutations in non-small-cell lung cancer.. Lancet Oncol.

[11] da Cunha Santos G, Shepherd FA, Tsao MS (2011). EGFR mutations and lung cancer.. Annu Rev Pathol.

[12] Wong MP, Lam WK, Wang E, Chiu SW, Lam CL (2002). Primary adenocarcinomas of the lung in nonsmokers show a distinct pattern of allelic imbalance.. Cancer Res.

[13] Wong MP, Fung LF, Wang E, Chow WS, Chiu SW (2003). Chromosomal aberrations of primary lung adenocarcinomas in nonsmokers.. Cancer.

[14] Powell CA, Bueno R, Borczuk AC, Caracta CF, Richards WG (2003). Patterns of allelic loss differ in lung adenocarcinomas of smokers and nonsmokers.. Lung Cancer.

[15] Albertson DG, Collins C, McCormick F, Gray JW (2003). Chromosome aberrations in solid tumors.. Nat Genet.

[16] Rhead B, Karolchik D, Kuhn RM, Hinrichs AS, Zweig AS (2010). The UCSC Genome Browser database: update 2010.. Nucleic Acids Res.

[17] Coe BP, Lockwood WW, Girard L, Chari R, Macaulay C (2006). Differential disruption of cell cycle pathways in small cell and non-small cell lung cancer.. Br J Cancer.

[18] Coe BP, Ylstra B, Carvalho B, Meijer GA, Macaulay C (2007). Resolving the resolution of array CGH.. Genomics.

[19] Beroukhim R, Getz G, Nghiemphu L, Barretina J, Hsueh T (2007). Assessing the significance of chromosomal aberrations in cancer: methodology and application to glioma.. Proc Natl Acad Sci U S A.

[20] Chitale D, Gong Y, Taylor BS, Broderick S, Brennan C (2009). An integrated genomic analysis of lung cancer reveals loss of DUSP4 in EGFR-mutant tumors.. Oncogene.

[21] Coe BP, Chari R, MacAulay C, Lam WL (2010). FACADE: a fast and sensitive algorithm for the segmentation and calling of high resolution array CGH data.. Nucleic Acids Res.

[22] Weir BA, Woo MS, Getz G, Perner S, Ding L (2007). Characterizing the cancer genome in lung adenocarcinoma.. Nature.

[23] Broet P, Tan P, Alifano M, Camilleri-Broet S, Richardson S (2009). Finding exclusively deleted or amplified genomic areas in lung adenocarcinomas using a novel chromosomal pattern analysis.. BMC Med Genomics.

[24] Newnham GM, Conron M, McLachlan S, Dobrovic A, Do H (2011). Integrated mutation, copy number and expression profiling in resectable non-small cell lung cancer.. BMC Cancer.

[25] Soh J, Okumura N, Lockwood WW, Yamamoto H, Shigematsu H (2009). Oncogene mutations, copy number gains and mutant allele specific imbalance (MASI) frequently occur together in tumor cells.. PLoS One.

[26] Rudin CM, Avila-Tang E, Harris CC, Herman JG, Hirsch FR (2009). Lung cancer in never smokers: molecular profiles and therapeutic implications.. Clin Cancer Res.

[27] Samet JM, Avila-Tang E, Boffetta P, Hannan LM, Olivo-Marston S (2009). Lung cancer in never smokers: clinical epidemiology and environmental risk factors.. Clin Cancer Res.

[28] Campbell JM, Lockwood WW, Buys TP, Chari R, Coe BP (2008). Integrative genomic and gene expression analysis of chromosome 7 identified novel oncogene loci in non-small cell lung cancer.. Genome.

[29] Bowtell DD (2010). The genesis and evolution of high-grade serous ovarian cancer.. Nat Rev Cancer.

