# Supplementary figures and images for: Lung Adenocarcinoma of Never Smokers and Smokers Harbor Differential Regions of Genetic Alteration and Exhibit Different Levels of Genomic Instability
Source: PLoS One. 2012 Mar 7;7(3):e33003. doi: 10.1371/journal.pone.0033003 (PMC3296775; doi:10.1371/journal.pone.0033003)

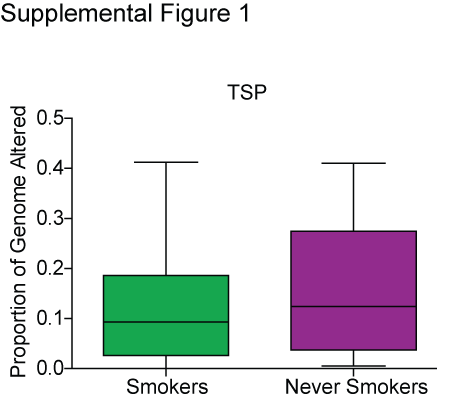

Supplement: Figure S1 — PGA in smokers and NS in the TSP dataset. Although it did not meet statistical significance, NS lung tumors have greater PGA than smoker lung tumors on average. (TIF) [file pone.0033003.s001.tif]

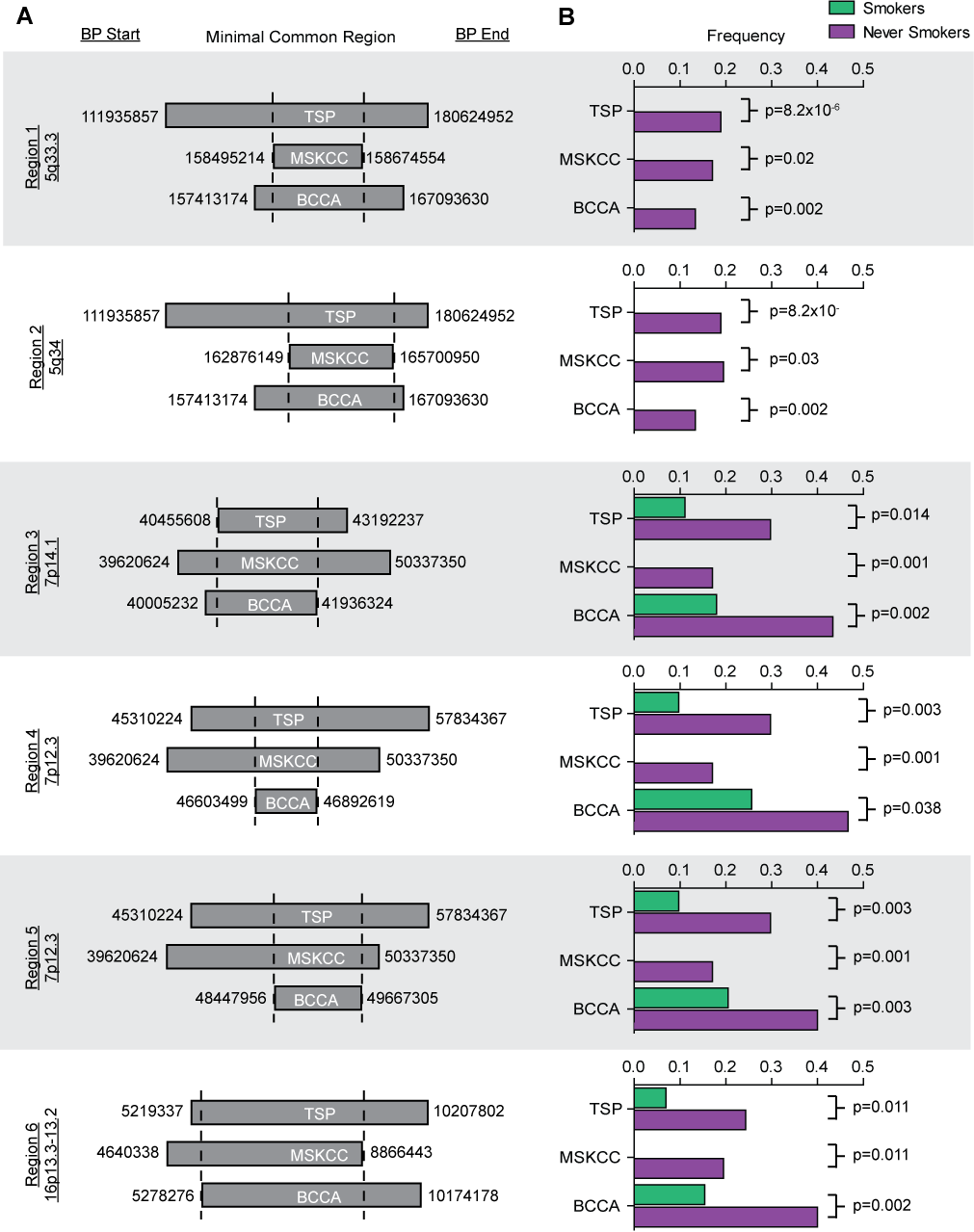

Supplement: Figure S2 — Six minimal common regions (MCRs) of difference between smokers and NS. The six regions described in Table 4 are illustrated here. The region from each dataset involved in the MCR is shown with the genomic coordinates flanking each region (A). Hashed lines indicate the MCR region boundaries. Regions are not drawn to scale. The frequencies of DNA copy number gains in smoker and NS tumors for each dataset is indicated (B). Since the differentially altered regions in each dataset were defined by merging adjacent significant regions into one (as described in the Methods), the frequencies illustrated are the minimum frequencies observed for regions contributing to the merged region. Fisher's exact test p-values for the comparison of alteration frequencies in NS and smokers are indicated for each region in each dataset. (TIF) [file pone.0033003.s002.tif]
