# Supplementary material for: Lung Adenocarcinoma of Never Smokers and Smokers Harbor Differential Regions of Genetic Alteration and Exhibit Different Levels of Genomic Instability
Source: PLoS One. 2012 Mar 7;7(3):e33003. doi: 10.1371/journal.pone.0033003 (PMC3296775; doi:10.1371/journal.pone.0033003)
Supplement: Table S1 — Genomic DNA PCR primers. PCR on genomic DNA was performed to determine EGFR and KRAS mutation status in the BCCA lung tumor cohort. Mutations and primer sequences are shown. (DOC) [file pone.0033003.s003.doc]

Table S1. Genomic DNA PCR primers.

| Amplicon | Forward Sequence | Reverse Sequence |
| --- | --- | --- |
| *EGFR* exon 19 | 5’ CCAGATCACTGGGCAGCATGTGGCACC 3’ | 5’ AGCAGGGTCTAGAGCAGAGCAGCTGCC 3’ |
| *EGFR* exon 21 | 5’ TCAGAGCCTGGCATGAACATGACCCTG 3’ | 5’ GGTCCCTGGTGTCAGGAAAATGCTGG 3’ |
| *KRAS* exon 2 | 5’ GTATTAACCTTATGTGTGACA 3’ | 5’ GTCCTGCACCAGTAATATGC 3’ |
