# Supplementary material for: Lung Adenocarcinoma of Never Smokers and Smokers Harbor Differential Regions of Genetic Alteration and Exhibit Different Levels of Genomic Instability
Source: PLoS One. 2012 Mar 7;7(3):e33003. doi: 10.1371/journal.pone.0033003 (PMC3296775; doi:10.1371/journal.pone.0033003)
Supplement: Table S2 — Matrix summarizing correlations between clinical and genetic variables in the 69 BCCA tumors. A correlation analysis was performed to determine the associations between different clinical and molecular factors in the BCCA lung tumor cohort. Correlation coefficients for each pair of variables are shown. (DOC) [file pone.0033003.s004.doc]

Table S2. Matrix summarizing correlations between clinical and genetic variables in the 69 BCCA tumors.

|  | Stage | Gender | Age | Smoking | EGFR | KRAS | RACE |
| --- | --- | --- | --- | --- | --- | --- | --- |
| Stage | 1.0000 | -0.0182 | -0.2393 | -0.0226 | 0.1075 | -0.0246 | -0.0911 |
| Gender |  | 1.0000 | 0.0029 | 0.0550 | 0.0980 | -0.1382 | 0.0000 |
| Age |  |  | 1.0000 | -0.1641 | 0.1382 | -0.2090 | 0.1260 |
| Smoking |  |  |  | 1.0000 | -0.6108 | 0.5235 | -0.6822 |
| *EGFR* |  |  |  |  | 1.0000 | -0.4763 | 0.6301 |
| *KRAS* |  |  |  |  |  | 1.0000 | -0.4410 |
| RACE |  |  |  |  |  |  | 1.0000 |
