# Supplementary material for: Lung Adenocarcinoma of Never Smokers and Smokers Harbor Differential Regions of Genetic Alteration and Exhibit Different Levels of Genomic Instability
Source: PLoS One. 2012 Mar 7;7(3):e33003. doi: 10.1371/journal.pone.0033003 (PMC3296775; doi:10.1371/journal.pone.0033003)
Supplement: Table S3 — Summary of alteration frequencies in BCCA smoker (n = 39) and NS (n = 30) lung tumors. The frequency of copy number alterations throughout the genome (calculated using a moving average window of 500 SNP array probes) was determined and summarized for each group (all 69 tumors, 39 smoker tumors and 30 never smoker tumors). The minimum (Min), maximum (Max), median, and average frequencies for each group are indicated. Total frequency (Gain & Loss), frequency of gain, and frequency of loss are reported. (DOC) [file pone.0033003.s005.doc]

Table S3. Summary of alteration frequencies in BCCA smoker (n=39) and NS (n=30) lung tumors.

| Group | Min | Max | Median | Average |
| --- | --- | --- | --- | --- |
| All Gain & Loss | 0 | 0.587594 | 0.160522 | 0.179258 |
| All Gain | 0.006551 | 0.587594 | 0.154174 | 0.184736 |
| All Loss | 0 | 0.466464 | 0.170464 | 0.17378 |
| Smokers Gain & Loss | 0 | 0.699179 | 0.137846 | 0.159058 |
| Smokers Gain | 0 | 0.699179 | 0.130821 | 0.166692 |
| Smokers Loss | 0 | 0.458974 | 0.150667 | 0.151425 |
| Never Smokers Gain & Loss | 0 | 0.609533 | 0.187133 | 0.205517 |
| Never Smokers Gain | 0.001933 | 0.609533 | 0.1904 | 0.208194 |
| Never Smoker Loss | 0 | 0.566667 | 0.183467 | 0.20284 |
