# Supplementary material for: Lung Adenocarcinoma of Never Smokers and Smokers Harbor Differential Regions of Genetic Alteration and Exhibit Different Levels of Genomic Instability
Source: PLoS One. 2012 Mar 7;7(3):e33003. doi: 10.1371/journal.pone.0033003 (PMC3296775; doi:10.1371/journal.pone.0033003)
Supplement: Table S4 — Multifactor ANOVA test results for assessing the effects of clinical and genetic factors on observed PGA in 69 BCCA tumors. A multifactor ANOVA was performed to investigate the effects of multiple factors on PGA in the BCCA lung tumor cohort (n = 69). The ANOVA test statistics are shown. (DOC) [file pone.0033003.s006.doc]

**Table S4. Multifactor ANOVA** test results for assessing the effects of clinical and genetic factors on observed PGA in 69 BCCA tumors.

| Variable | Df | SumSq | MeanSq | Fvalue | Pr(>F) |
| --- | --- | --- | --- | --- | --- |
| Stage | 3 | 0.08302 | 0.027674 | 0.7237 | 0.54185 |
| Gender | 1 | 0.00512 | 0.005124 | 0.134 | 0.71561 |
| Age | 1 | 0.02771 | 0.027708 | 0.7246 | 0.39807 |
| **Smoking** | **1** | **0.13933** | **0.139329** | **3.6438** | **0.06115** |
| *EGFR* | 1 | 0.09534 | 0.095341 | 2.4934 | 0.11967 |
| *KRAS* | 1 | 0.00581 | 0.005811 | 0.152 | 0.69805 |
| RACE | 1 | 0.02434 | 0.024336 | 0.6364 | 0.4282 |
| Residuals | 59 | 2.25601 | 0.038237 |  |  |
