# Supplementary material for: Lung Adenocarcinoma of Never Smokers and Smokers Harbor Differential Regions of Genetic Alteration and Exhibit Different Levels of Genomic Instability
Source: PLoS One. 2012 Mar 7;7(3):e33003. doi: 10.1371/journal.pone.0033003 (PMC3296775; doi:10.1371/journal.pone.0033003)
Supplement: Table S5 — Multifactor ANOVA test results for assessing the effects of clinical and genetic factors on observed PGA in NS tumors only. A multifactor ANOVA was performed to investigate the effects of multiple factors on PGA in never smokers only (n = 30). The ANOVA test statistics are shown. (DOC) [file pone.0033003.s007.doc]

**Table S5. Multifactor ANOVA test results for assessing the effects of clinical and genetic factors on observed PGA in NS tumors only.**

| Variable | Df | Sum S | Mean Sq | F value | Pr(>F) |
| --- | --- | --- | --- | --- | --- |
| Stage | 2 | 0.08184 | 0.040922 | 1.283 | 0.2971 |
| Gender | 1 | 0.0295 | 0.029497 | 0.9248 | 0.3467 |
| Age | 1 | 0.03218 | 0.032181 | 1.009 | 0.3261 |
| ***EGFR*** | **1** | **0.10263** | **0.102628** | **3.2177** | **0.0866** |
| *KRAS* | 1 | 0.06582 | 0.065819 | 2.0636 | 0.1649 |
| Race | 1 | 0.01545 | 0.015451 | 0.4844 | 0.4937 |
| Residuals | 22 | 0.70169 | 0.031895 |  |  |
