# Supplementary material for: Lung Adenocarcinoma of Never Smokers and Smokers Harbor Differential Regions of Genetic Alteration and Exhibit Different Levels of Genomic Instability
Source: PLoS One. 2012 Mar 7;7(3):e33003. doi: 10.1371/journal.pone.0033003 (PMC3296775; doi:10.1371/journal.pone.0033003)
Supplement: Table S6 — High-level DNA changes in smoker lung tumors. GISTIC was used to reveal high level DNA alterations in 39 smoker lung tumors from the BCCA. Copy number status, genomic location, and frequency of alteration are indicated. (DOC) [file pone.0033003.s008.doc]

Table S6. High-level DNA changes in smoker lung tumors.

| **Event** | **Cytoband** | **Chr** | **Start** | **End** | **Size (Mbp)** | **q-Value** | **Frequency of Alteration** | **Number of High Level Events** |
| --- | --- | --- | --- | --- | --- | --- | --- | --- |
| Amplification | 1p36.32 | chr1 | 826949 | 3736736 | 2.909787 | 0.0094601 | 0.13 | 5 |
| Amplification | 1p36.22 | chr1 | 10631135 | 10758380 | 0.127245 | 0.036592 | 0.13 | 5 |
| Amplification | 1p36.13 | chr1 | 16840799 | 16954998 | 0.114199 | 0.022618 | 0.15 | 5 |
| Amplification | 1p36.12 | chr1 | 21773032 | 21872612 | 0.09958 | 0.033815 | 0.13 | 5 |
| Amplification | 1q23.3 | chr1 | 155434696 | 164158848 | 8.724152 | 0.000014282 | 0.31 | 8 |
| Amplification | 1q31.2 | chr1 | 166768132 | 198043842 | 31.27571 | 0.00002427 | 0.28 | 11 |
| Amplification | 1q41 | chr1 | 215319993 | 222444278 | 7.124285 | 0.0030607 | 0.18 | 7 |
| Amplification | 1q44 | chr1 | 242952015 | 246921583 | 3.969568 | 0.0022915 | 0.21 | 7 |
| Amplification | 2q33.3 | chr2 | 208929826 | 208955226 | 0.0254 | 0.036592 | 0.10 | 4 |
| Amplification | 2q35 | chr2 | 220030718 | 220135240 | 0.104522 | 0.036592 | 0.13 | 5 |
| Amplification | 2q37.3 | chr2 | 239593290 | 239770150 | 0.17686 | 0.04583 | 0.13 | 4 |
| Amplification | 4p16.3 | chr4 | 590273 | 2361186 | 1.770913 | 0.028921 | 0.13 | 4 |
| Amplification | 5p15.33 | chr5 | 1 | 12107755 | 12.107754 | 0.000014282 | 0.26 | 9 |
| Amplification | 5q31.3 | chr5 | 140141350 | 140617729 | 0.476379 | 0.0028584 | 0.13 | 5 |
| Amplification | 5q35.3 | chr5 | 177587657 | 179539993 | 1.952336 | 0.0034824 | 0.18 | 7 |
| Amplification | 6p25.3 | chr6 | 329547 | 405050 | 0.075503 | 0.049934 | 0.13 | 5 |
| Amplification | 6p25.2 | chr6 | 2788612 | 3077541 | 0.288929 | 0.030448 | 0.15 | 6 |
| Amplification | 6q27 | chr6 | 170173627 | 170453855 | 0.280228 | 0.036592 | 0.15 | 5 |
| Amplification | 7p22.1 | chr7 | 4046414 | 6743582 | 2.697168 | 0.00013588 | 0.21 | 8 |
| Amplification | 7p21.3 | chr7 | 12446458 | 13980851 | 1.534393 | 0.0015353 | 0.21 | 7 |
| Amplification | 7p15.2 | chr7 | 27089103 | 27264315 | 0.175212 | 0.000033934 | 0.21 | 8 |
| Amplification | 7p13 | chr7 | 44038387 | 44348551 | 0.310164 | 0.0074015 | 0.18 | 7 |
| Amplification | 7q11.21 | chr7 | 57940128 | 62098219 | 4.158091 | 0.000014282 | 0.23 | 9 |
| Amplification | 8p23.3 | chr8 | 569115 | 2181333 | 1.612218 | 0.0004602 | 0.21 | 7 |
| Amplification | 8q11.1 | chr8 | 43558072 | 47921143 | 4.363071 | 0.0001499 | 0.21 | 6 |
| Amplification | 8q11.23 | chr8 | 55522879 | 55560610 | 0.037731 | 0.0066126 | 0.15 | 5 |
| Amplification | 8q24.3 | chr8 | 140684470 | 146027170 | 5.3427 | 0.00017141 | 0.21 | 7 |
| Amplification | 9p21.3 | chr9 | 21939762 | 22005433 | 0.065671 | 0.00004419 | 0.21 | 7 |
| Amplification | 9q22.32 | chr9 | 97299012 | 97323995 | 0.024983 | 0.014301 | 0.13 | 5 |
| Amplification | 9q33.3 | chr9 | 125796462 | 125850663 | 0.054201 | 0.004203 | 0.15 | 6 |
| Amplification | 10p15.2 | chr10 | 3143204 | 3180796 | 0.037592 | 0.01538 | 0.13 | 4 |
| Amplification | 10q21.1 | chr10 | 58749758 | 58775016 | 0.025258 | 0.0021521 | 0.13 | 5 |
| Amplification | 10q26.13 | chr10 | 126184491 | 126326147 | 0.141656 | 0.047216 | 0.10 | 4 |
| Amplification | 11p15.5 | chr11 | 252270 | 3150503 | 2.898233 | 0.00035635 | 0.18 | 6 |
| Amplification | 11p11.2 | chr11 | 48620184 | 48848452 | 0.228268 | 0.0083986 | 0.15 | 6 |
| Amplification | 11q13.1 | chr11 | 63496145 | 70976822 | 7.480677 | 0.0022915 | 0.18 | 6 |
| Amplification | 12q13.13 | chr12 | 52577813 | 52748743 | 0.17093 | 0.00059989 | 0.21 | 8 |
| Amplification | 12q14.1 | chr12 | 56279758 | 56456601 | 0.176843 | 0.01768 | 0.18 | 5 |
| Amplification | 12q24.31 | chr12 | 123361104 | 123554310 | 0.193206 | 0.047216 | 0.13 | 4 |
| Amplification | 12q24.33 | chr12 | 129473409 | 131997175 | 2.523766 | 0.033815 | 0.13 | 4 |
| Amplification | 13q12.11 | chr13 | 19606560 | 19658247 | 0.051687 | 0.035238 | 0.13 | 4 |
| Amplification | 13q34 | chr13 | 109893872 | 113955243 | 4.061371 | 0.0016669 | 0.18 | 6 |
| Amplification | 16p13.3 | chr16 | 243858 | 3145335 | 2.901477 | 0.037195 | 0.13 | 4 |
| Amplification | 16p11.2 | chr16 | 29021403 | 29148931 | 0.127528 | 0.026558 | 0.15 | 5 |
| Amplification | 16q24.2 | chr16 | 85080401 | 88625248 | 3.544847 | 0.036592 | 0.13 | 5 |
| Amplification | 17q21.33 | chr17 | 45290824 | 46061022 | 0.770198 | 0.0038913 | 0.18 | 7 |
| Amplification | 17q25.1 | chr17 | 68642169 | 78643087 | 10.000918 | 0.00016317 | 0.21 | 7 |
| Amplification | 18q23 | chr18 | 74819218 | 75791418 | 0.9722 | 0.028312 | 0.13 | 5 |
| Amplification | 19p13.3 | chr19 | 316051 | 5967999 | 5.651948 | 0.036592 | 0.13 | 4 |
| Amplification | 19q12 | chr19 | 24165484 | 32819473 | 8.653989 | 0.000014282 | 0.26 | 9 |
| Amplification | 19q13.2 | chr19 | 43329204 | 47614346 | 4.285142 | 0.00067538 | 0.23 | 7 |
| Amplification | 20p13 | chr20 | 3586111 | 3756353 | 0.170242 | 0.016378 | 0.15 | 6 |
| Amplification | 20q13.13 | chr20 | 48562749 | 49586304 | 1.023555 | 0.033815 | 0.15 | 6 |
| Amplification | 20q13.33 | chr20 | 59283093 | 62426596 | 3.143503 | 0.0044698 | 0.15 | 6 |
| Amplification | 22q13.1 | chr22 | 35595026 | 38428789 | 2.833763 | 0.003703 | 0.18 | 6 |
| Deletion | 1p36.32 | chr1 | 2713206 | 3708916 | 0.99571 | 0.049878 | 0.15 | 1 |
| Deletion | 1p21.1 | chr1 | 102592549 | 102610716 | 0.018167 | 0.028082 | 0.18 | 1 |
| Deletion | 2p24.3 | chr2 | 14263243 | 14281096 | 0.017853 | 0.023866 | 0.18 | 0 |
| Deletion | 2p16.1 | chr2 | 57244588 | 57280053 | 0.035465 | 0.039856 | 0.15 | 1 |
| Deletion | 2q31.1 | chr2 | 169500668 | 169542021 | 0.041353 | 0.0057133 | 0.18 | 1 |
| Deletion | 3p26.1 | chr3 | 7062003 | 7126263 | 0.06426 | 0.020701 | 0.18 | 0 |
| Deletion | 3p24.2 | chr3 | 25583581 | 25606311 | 0.02273 | 0.0071581 | 0.21 | 0 |
| Deletion | 3p12.3 | chr3 | 76792826 | 78564868 | 1.772042 | 0.039056 | 0.15 | 1 |
| Deletion | 3q13.11 | chr3 | 104825688 | 104903585 | 0.077897 | 0.042149 | 0.15 | 0 |
| Deletion | 3q13.13 | chr3 | 111616695 | 111690272 | 0.073577 | 0.0060628 | 0.18 | 1 |
| Deletion | 4p16.2 | chr4 | 3379182 | 3520414 | 0.141232 | 0.025749 | 0.18 | 1 |
| Deletion | 4q13.1 | chr4 | 63422139 | 63516496 | 0.094357 | 0.023866 | 0.15 | 1 |
| Deletion | 4q13.3 | chr4 | 72549934 | 72671419 | 0.121485 | 0.022284 | 0.18 | 1 |
| Deletion | 4q28.3 | chr4 | 138146913 | 138162344 | 0.015431 | 0.042149 | 0.15 | 0 |
| Deletion | 5q13.3 | chr5 | 75273943 | 75332657 | 0.058714 | 0.001742 | 0.21 | 2 |
| Deletion | 5q14.3 | chr5 | 88571589 | 89309321 | 0.737732 | 0.019087 | 0.18 | 1 |
| Deletion | 5q21.1 | chr5 | 100733754 | 100794702 | 0.060948 | 0.001742 | 0.21 | 2 |
| Deletion | 5q23.2 | chr5 | 125245606 | 125352370 | 0.106764 | 0.0035807 | 0.21 | 0 |
| Deletion | 5q31.3 | chr5 | 140503546 | 140610282 | 0.106736 | 0.034846 | 0.15 | 1 |
| Deletion | 5q33.2 | chr5 | 154817253 | 154854084 | 0.036831 | 0.049686 | 0.15 | 1 |
| Deletion | 6q12 | chr6 | 67179217 | 68454950 | 1.275733 | 0.002863 | 0.23 | 0 |
| Deletion | 6q16.1 | chr6 | 94527588 | 94644289 | 0.116701 | 0.030335 | 0.18 | 0 |
| Deletion | 6q22.31 | chr6 | 121037193 | 121136348 | 0.099155 | 0.017893 | 0.21 | 0 |
| Deletion | 6q27 | chr6 | 166740890 | 168829383 | 2.088493 | 0.019087 | 0.21 | 0 |
| Deletion | 7p22.2 | chr7 | 1596895 | 2732453 | 1.135558 | 0.029976 | 0.18 | 1 |
| Deletion | 7p21.3 | chr7 | 11658130 | 11755316 | 0.097186 | 0.0084418 | 0.21 | 0 |
| Deletion | 7q11.21 | chr7 | 61484091 | 62091130 | 0.607039 | 0.042149 | 0.15 | 1 |
| Deletion | 7q21.13 | chr7 | 89464548 | 89532619 | 0.068071 | 0.020701 | 0.18 | 1 |
| Deletion | 8p22 | chr8 | 13696094 | 13725542 | 0.029448 | 0.020701 | 0.18 | 0 |
| Deletion | 8p11.1 | chr8 | 43558072 | 43943204 | 0.385132 | 0.001742 | 0.18 | 3 |
| Deletion | 8q12.1 | chr8 | 61124474 | 61170287 | 0.045813 | 0.0071581 | 0.21 | 0 |
| Deletion | 9p23 | chr9 | 9394585 | 10575477 | 1.180892 | 0.001937 | 0.26 | 0 |
| Deletion | 9p22.2 | chr9 | 17668088 | 18191550 | 0.523462 | 0.003002 | 0.21 | 0 |
| Deletion | 9p21.3 | chr9 | 21944954 | 22008780 | 0.063826 | 0.001742 | 0.21 | 1 |
| Deletion | 9p21.1 | chr9 | 30192335 | 31709066 | 1.516731 | 0.003002 | 0.23 | 0 |
| Deletion | 10q21.1 | chr10 | 58742794 | 58775016 | 0.032222 | 0.003002 | 0.21 | 2 |
| Deletion | 10q23.31 | chr10 | 91941310 | 92040888 | 0.099578 | 0.045615 | 0.18 | 0 |
| Deletion | 10q25.1 | chr10 | 109115788 | 109619108 | 0.50332 | 0.039856 | 0.18 | 0 |
| Deletion | 10q26.3 | chr10 | 133708154 | 134748055 | 1.039901 | 0.029976 | 0.18 | 1 |
| Deletion | 11p15.4 | chr11 | 2686540 | 3134514 | 0.447974 | 0.039056 | 0.15 | 1 |
| Deletion | 11p14.1 | chr11 | 28031587 | 28225293 | 0.193706 | 0.049878 | 0.13 | 1 |
| Deletion | 11p11.12 | chr11 | 50425555 | 50593832 | 0.168277 | 0.039856 | 0.15 | 1 |
| Deletion | 11q14.3 | chr11 | 87251347 | 88115585 | 0.864238 | 0.014341 | 0.18 | 1 |
| Deletion | 11q14.3 | chr11 | 90585766 | 91511648 | 0.925882 | 0.012348 | 0.21 | 0 |
| Deletion | 13q31.1 | chr13 | 82684125 | 82737085 | 0.05296 | 0.0057133 | 0.18 | 1 |
| Deletion | 14q11.2 | chr14 | 21412283 | 21460458 | 0.048175 | 0.042149 | 0.13 | 2 |
| Deletion | 15q21.1 | chr15 | 44160130 | 45288548 | 1.128418 | 0.042149 | 0.18 | 0 |
| Deletion | 16q21 | chr16 | 59805340 | 59920394 | 0.115054 | 0.016418 | 0.21 | 0 |
| Deletion | 18q21.1 | chr18 | 43649369 | 43664649 | 0.01528 | 0.039856 | 0.15 | 1 |
| Deletion | 19p12 | chr19 | 24163308 | 32883874 | 8.720566 | 0.001937 | 0.21 | 2 |
| Deletion | 21q22.3 | chr21 | 43578866 | 44827398 | 1.248532 | 0.020701 | 0.18 | 1 |
| Deletion | 22q13.31 | chr22 | 44424566 | 44631074 | 0.206508 | 0.015305 | 0.18 | 1 |
