# Supplementary material for: Lung Adenocarcinoma of Never Smokers and Smokers Harbor Differential Regions of Genetic Alteration and Exhibit Different Levels of Genomic Instability
Source: PLoS One. 2012 Mar 7;7(3):e33003. doi: 10.1371/journal.pone.0033003 (PMC3296775; doi:10.1371/journal.pone.0033003)
Supplement: Table S7 — High-level DNA changes in NS lung tumors. GISTIC was used to reveal high level DNA alterations in 30 never smoker lung tumors from the BCCA. Copy number status, genomic location, and frequency of alteration are indicated. (DOC) [file pone.0033003.s009.doc]

Table S7. High-level DNA changes in NS lung tumors.

| **Event** | **Cytoband** | **Chr** | **Start** | **End** | **Size (Mbp)** | **q-Value** | **Frequency of Alteration** | **Number of High Level Events** |
| --- | --- | --- | --- | --- | --- | --- | --- | --- |
| Amplification | 5p15.32 | chr5 | 4395313 | 4778404 | 0.383091 | 0.047723 | 0.23 | 5 |
| Amplification | 7q11.21 | chr7 | 57940128 | 62094909 | 4.154781 | 0.0013223 | 0.27 | 7 |
| Amplification | 8p11.1 | chr8 | 43899453 | 43943204 | 0.043751 | 0.040828 | 0.20 | 6 |
| Amplification | 19q12 | chr19 | 24256798 | 32779216 | 8.522418 | 0.0089815 | 0.23 | 6 |
| Amplification | 20q13.33 | chr20 | 59185038 | 62426596 | 3.241558 | 0.041743 | 0.20 | 5 |
| Deletion | 1p31.1 | chr1 | 74391693 | 74506730 | 0.115037 | 0.0017076 | 0.30 | 1 |
| Deletion | 1p22.3 | chr1 | 86164664 | 86184880 | 0.020216 | 0.02429 | 0.20 | 2 |
| Deletion | 2q32.1 | chr2 | 184849279 | 184921951 | 0.072672 | 0.012604 | 0.27 | 1 |
| Deletion | 2q33.3 | chr2 | 208925237 | 208990371 | 0.065134 | 0.000018815 | 0.30 | 3 |
| Deletion | 3p24.2 | chr3 | 24470944 | 24567811 | 0.096867 | 0.0017076 | 0.30 | 1 |
| Deletion | 3p22.3 | chr3 | 35469901 | 35999377 | 0.529476 | 0.0053625 | 0.30 | 0 |
| Deletion | 3p12.3 | chr3 | 76548884 | 77681632 | 1.132748 | 0.000054826 | 0.37 | 2 |
| Deletion | 3q12.1 | chr3 | 99922328 | 100216733 | 0.294405 | 0.0019805 | 0.33 | 1 |
| Deletion | 3q23 | chr3 | 144238350 | 144336633 | 0.098283 | 0.0043363 | 0.23 | 3 |
| Deletion | 3q26.1 | chr3 | 167424967 | 167479979 | 0.055012 | 0.011027 | 0.27 | 0 |
| Deletion | 3q27.1 | chr3 | 185685726 | 185781860 | 0.096134 | 0.013451 | 0.23 | 1 |
| Deletion | 3q28 | chr3 | 191700938 | 191721841 | 0.020903 | 0.028536 | 0.23 | 1 |
| Deletion | 4p16.2 | chr4 | 3186614 | 3520414 | 0.3338 | 0.011146 | 0.27 | 1 |
| Deletion | 4p15.1 | chr4 | 28562973 | 28593695 | 0.030722 | 0.044763 | 0.23 | 0 |
| Deletion | 4q13.1 | chr4 | 65154749 | 65197754 | 0.043005 | 0.0036492 | 0.27 | 2 |
| Deletion | 5q15 | chr5 | 95262172 | 95266620 | 0.004448 | 0.043064 | 0.27 | 0 |
| Deletion | 5q31.3 | chr5 | 140141350 | 140614343 | 0.472993 | 0.0043363 | 0.23 | 1 |
| Deletion | 6q12 | chr6 | 67706678 | 67758736 | 0.052058 | 0.037062 | 0.23 | 1 |
| Deletion | 6q16.1 | chr6 | 94018754 | 95135848 | 1.117094 | 0.0020575 | 0.30 | 1 |
| Deletion | 7p15.2 | chr7 | 27087010 | 27262142 | 0.175132 | 0.0017076 | 0.20 | 4 |
| Deletion | 8p23.3 | chr8 | 585909 | 1935822 | 1.349913 | 0.031751 | 0.23 | 1 |
| Deletion | 8p12 | chr8 | 33437510 | 33536672 | 0.099162 | 0.019774 | 0.27 | 0 |
| Deletion | 8q24.3 | chr8 | 143307535 | 146032498 | 2.724963 | 0.047884 | 0.23 | 1 |
| Deletion | 9p24.1 | chr9 | 4641983 | 5448034 | 0.806051 | 0.000054826 | 0.30 | 4 |
| Deletion | 9p21.3 | chr9 | 20538755 | 28805650 | 8.266895 | 4.0417E-16 | 0.53 | 2 |
| Deletion | 9p13.2 | chr9 | 36873045 | 38526474 | 1.653429 | 0.038578 | 0.23 | 1 |
| Deletion | 9q22.2 | chr9 | 90963297 | 91053153 | 0.089856 | 0.033257 | 0.23 | 1 |
| Deletion | 9q31.1 | chr9 | 105630708 | 106428489 | 0.797781 | 5.8086E-06 | 0.43 | 1 |
| Deletion | 9q31.3 | chr9 | 113414551 | 113473890 | 0.059339 | 0.015866 | 0.17 | 4 |
| Deletion | 9q33.3 | chr9 | 125793041 | 126683885 | 0.890844 | 0.0081088 | 0.23 | 3 |
| Deletion | 10q21.1 | chr10 | 58736845 | 58775582 | 0.038737 | 2.1563E-07 | 0.37 | 3 |
| Deletion | 10q23.32 | chr10 | 92897044 | 92921271 | 0.024227 | 0.0014686 | 0.20 | 4 |
| Deletion | 11q14.1 | chr11 | 80673268 | 81579681 | 0.906413 | 0.0015983 | 0.33 | 0 |
| Deletion | 11q14.3 | chr11 | 89855999 | 90686553 | 0.830554 | 0.0033945 | 0.30 | 0 |
| Deletion | 11q22.3 | chr11 | 103732139 | 103815119 | 0.08298 | 0.0017076 | 0.33 | 0 |
| Deletion | 13q12.11 | chr13 | 19924494 | 19976915 | 0.052421 | 0.026387 | 0.23 | 1 |
| Deletion | 13q21.1 | chr13 | 53725815 | 55125876 | 1.400061 | 0.0092921 | 0.30 | 0 |
| Deletion | 15q26.3 | chr15 | 98175569 | 98233096 | 0.057527 | 0.0294 | 0.27 | 0 |
| Deletion | 16q24.1 | chr16 | 85078079 | 85182555 | 0.104476 | 0.0017076 | 0.30 | 2 |
| Deletion | 17p11.2 | chr17 | 17511553 | 17693878 | 0.182325 | 0.033257 | 0.23 | 0 |
| Deletion | 18p11.32 | chr18 | 2829146 | 2902919 | 0.073773 | 0.028044 | 0.23 | 1 |
| Deletion | 18q12.3 | chr18 | 33686994 | 40305262 | 6.618268 | 0.0018373 | 0.33 | 0 |
| Deletion | 18q23 | chr18 | 72180559 | 75198347 | 3.017788 | 0.0017076 | 0.30 | 1 |
| Deletion | 19q12 | chr19 | 24299013 | 32762038 | 8.463025 | 0.016729 | 0.27 | 0 |
| Deletion | 22q13.31 | chr22 | 44621024 | 45245124 | 0.6241 | 0.015866 | 0.27 | 1 |
