# Supplementary material for: Lung Adenocarcinoma of Never Smokers and Smokers Harbor Differential Regions of Genetic Alteration and Exhibit Different Levels of Genomic Instability
Source: PLoS One. 2012 Mar 7;7(3):e33003. doi: 10.1371/journal.pone.0033003 (PMC3296775; doi:10.1371/journal.pone.0033003)
Supplement: Table S8 — Matrix summarizing correlations between each minimal common region identified in the 69 BCCA tumors. The six minimal common regions identified in the BCCA tumor cohort were assessed for their correlations with one another. The correlation coefficients for each pair of regions are shown. (DOC) [file pone.0033003.s010.doc]

**Table S8**. Matrix summarizing correlations between each minimal common region identified in the 69 BCCA tumors.

| Region | 5q33.3 | 5q34 | 7p14.1 | 7p12.3 – 1 | 7p12.3 - 2 | 16p13.3-13.2 |
| --- | --- | --- | --- | --- | --- | --- |
| 5q33.3 | 1 | 0.643614 | 0.3577756 | 0.3032838 | 0.2208537 | 0.5160225 |
| 5q34 |  | 1 | 0.3879725 | 0.2314931 | 0.3706458 | 0.3879725 |
| 7p14.1 |  |  | 1 | 0.8176294 | 0.8297814 | 0.2959184 |
| 7p12.3 - 1 |  |  |  | 1 | 0.7828714 | 0.3118587 |
| 7p12.3 - 2 |  |  |  |  | 1 | 0.2270981 |
| 16p13.3-13.2 |  |  |  |  |  | 1 |
